# Economic evaluation of increasing population rates of cardiac catheterization

**DOI:** 10.1186/1472-6963-11-324

**Published:** 2011-11-24

**Authors:** Fiona M Clement, William A Ghali, Stephane Rinfret, Braden J Manns

**Affiliations:** 1 Department of Medicine, Faculty of Medicine, University of Calgary, Foothills Medical Centre - North Tower, 9th Floor, 1403 - 29th Street NW, Calgary, AB T2N 2T9, Canada; 2 Department of Community Health Sciences, Faculty of Medicine, University of Calgary, TRW Building 3rd Floor, 3280 Hospital Drive NW, Calgary, Alberta T2N 4Z6, Canada; 3 Centre for Health and Policy Studies, Faculty of Medicine, University of Calgary, TRW Building 3rd Floor, 3280 Hospital Drive NW, Calgary, Alberta T2N 4Z6, Canada; 4 Cardiology department, Laval Hospital- Laval University Heart and Lung Institute, 2725 Chemin Ste-Foy, Sainte-Foy, Quebec G1V 4G5 Canada

## Abstract

**Background:**

Increasing population rates of cardiac catheterization can lead to the detection of more people with high risk coronary disease and opportunity for subsequent revascularization. However, such a strategy should only be undertaken if it is cost-effective.

**Methods:**

Based on data from a cohort of patients undergoing cardiac catheterization, and efficacy data from clinical trials, we used a Markov model that considered 1) the yield of high-risk cases as the catheterization rate increases, 2) the long-term survival, quality of life and costs for patients with high risk disease, and 3) the impact of revascularization on survival, quality of life and costs. The cost per quality-adjusted life year was calculated overall, and by indication, age, and sex subgroups.

**Results:**

Increasing the catheterization rate was associated with a cost per QALY of CAN$26,470. The cost per QALY was most attractive in females with Acute Coronary Syndromes (ACS) ($20,320 per QALY gained), and for ACS patients over 75 years of age ($16,538 per QALY gained). However, there is significant model uncertainty associated with the efficacy of revascularization.

**Conclusion:**

A strategy of increasing cardiac catheterization rates among eligible patients is associated with a cost per QALY similar to that of other funded interventions. However, there is significant model uncertainty. A decision to increase population rates of catheterization requires consideration of the accompanying opportunity costs, and careful thought towards the most appropriate strategy.

## Background

Cardiac catheterization is a diagnostic or interventional procedure involving insertion of a catheter into a chamber or vessel of the heart. Once inserted contrast dye is injected and x-rays are taken to identify coronary stenoses within the heart [1]. One of the primary purposes of cardiac catheterization as an intervention is to identify patients with high-risk coronary artery disease, often defined as 3-vessel disease or left main disease. This is because revascularization in suitable patients with high-risk anatomy using percutaneous coronary intervention (PCI) or coronary artery bypass grafting (CABG) improves survival and quality of life [2-5].

Previous work has demonstrated a linear increase in the number of high-risk coronary artery disease cases detected when population catheterization rates vary across their demonstrated range [6]. Although increasing the population catheterization rate may detect more high risk cases, offering an opportunity to improve health outcomes through revascularization, any strategy that increases the use of coronary catheterization will also expose additional people to the small but well-known procedural risks of cardiac catheterization, as well as the more notable risk of subsequent PCI or CABG if performed. Furthermore, increasing the use of cardiac catheterization has the potential to incur additional expenditures for the health care system. Given the resource constraints of all health care systems, whether they are publicly or privately funded, there is a need to formally study the economic implications of implementing a policy designed to increase catheterization rates.

The Alberta Provincial Project for Outcomes Assessment in Coronary Heart (APPROACH) is a prospective cohort initiative that captures detailed clinical information on all patients undergoing cardiac catheterization within the province of Alberta, Canada (population approximately 3 million) [7]. APPROACH patients are followed longitudinally to assess clinical, economic and quality of life outcomes. This data resource provides a unique opportunity to conduct an economic evaluation of a policy designed to increase population cardiac catheterization rates. Using the APPROACH cohort, we estimated clinical event rates, health care costs, and health-related quality of life (HRQOL) for patients undergoing catheterization with or without subsequent PCI or CABG. By combining this information with the incremental yield of high-risk cases that would be detected by increasing population catheterization rates [6], and the results of randomized studies documenting the effectiveness of revascularization relative to medical management, in patients with high risk disease, we estimated the cost-effectiveness of increasing the population catheterization rate compared to maintaining the current rate.

## Methods

### Study design

We estimated the cost per quality-adjusted life year (QALY) gained for a strategy of increasing cardiac catheterization rates for patients potentially eligible to undergo catheterization compared to a contemporary population catheterization rate from Alberta, Canada (496 per 100,000, the rate seen in 2005), and subsequently for specific patient subgroups of interest where increasing catheterization rates might be considered. The subgroups of a priori interest included indication for catheterization (acute coronary syndrome (ACS) vs non-ACS), sex, and age. These are identified subgroups with differential benefit associated with revascularisation [8,9].

### Decision analytic model

**Figure 1:**
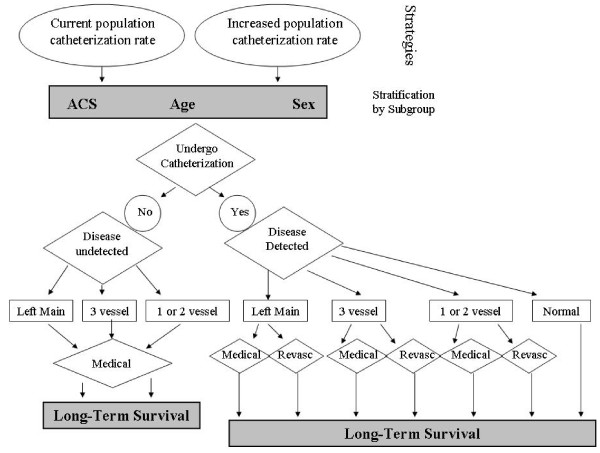
**Model structure**.

We used a Markov process to model the cost and clinical outcomes over a patient's lifetime for patients potentially eligible for catheterization in 1-year time intervals. A conceptual depiction of the model is presented in Figure 1. In broad terms, the model captures the possible events that can ensue when a patient potentially eligible for catheterization either undergoes cardiac catheterization (or not) in both ACS and non-ACS scenarios. Based on their coronary anatomy (left main disease, 3-vessel disease, 1-to-2 vessel disease, or normal/near normal coronaries), and consideration of both patient and provider preferences, patients are then treated medically or revascularized, assuming (for the comparator strategy) the existing revascularization rate that was observed in the APPROACH cohort for patients with left main disease, 3-vessel disease, 1-to-2 vessel disease, or normal/near normal coronaries. After undergoing catheterization and subsequent revascularization as appropriate, patients then have an on-going risk of death over their lifetime based on their indication for catheterization (ACS/non-ACS), age, sex, coronary anatomy, and treatment received. Patients undergoing cardiac catheterization who are found to have normal coronary arteries and have no further treatment are modelled in our Markov model to experience normal population based rates of annual mortality.

Among patients who do not undergo catheterization, a proportion will have undetected high-risk coronary artery disease - by definition, these patients receive medical management only. Some of these patients would have been eligible for revascularization had they undergone a catheterization, and thus the benefit of an increased catheterization rate is the additional detection of previously undetected patients with high-risk coronary anatomy who have the potential to benefit from revascularization, since randomized controlled trials have demonstrated a survival advantage with revascularization in this high-risk group [5]. Hence, in our analysis where we are modelling the effectiveness and cost-effectiveness of higher catheterization rates, the treatment strategies differ only by the size of this undetected group - when population catheterization rates are higher, the number of patients with high-risk coronary anatomy who are treated medically becomes smaller because more patients are detected and revascularized. By implication, the most important input variables in our analysis relate to the impact of revascularization in this patient subgroup on survival, quality of life and health care costs.

### Target Population

The target population of interest was all patients potentially eligible to undergo cardiac catheterization. An assumption inherent to our analysis is that the additional high risk cases detected among the eligible patients undergoing catheterization would be similar to the high risk cases detected using the current rate of catheterization. To test this assumption, patient demographics, intervention rates and outcomes were compared across all 9 health regions in Alberta, where age and sex adjusted catheterization rates varied more than 3-fold. When comparing the high-risk cases across tertiles of utilization, there were no differences in the survival and revascularization rates between regions, suggesting that there are few differences between detected and undetected high-risk cases.

### Comparators

The contemporary catheterization rate is based on the 2005 Alberta population catheterization rate taken from the APPROACH database (496 per 100,000) [10]. The increased catheterization rate strategy assumes that a greater proportion of eligible patients within a health region would receive cardiac catheterization. Given that the relationship between catheterization rate and high-risk disease yield has previously been shown to be linear, the cost per QALY calculated is reported per additional catheterization done. In-so-far as increasing the catheterization rate results in linearly increasing detection of high-risk cases [6], the cost per QALY gained by increasing catheterization rates can be assumed to be constant.

### Model Inputs

#### 1. Patient Cohorts (Table 1)

**Table 1:** Clinical outcomes and utility values, age and sex subgroup (N = 11527)*

	Males (n = 7885, 68%)			Females (n = 3642, 32%)		
	Under 65 n = 4684	65-75 n = 1992	Over 75 n = 1209	Under 65 n = 1722	65-75 n = 1038	Over 75 n = 882
Risk of death associated with catheterization (%)**	0.01	0.03	0.01	0.01	0.01	0.06
**Left main disease (%,n)**	**5.3 (247)**	**11.0 (220)**	**17.0 (205)**	**2.3 (40)**	**4.0 (41)**	**7.5 (66)**
Probability of revascularization (%)	84.2	81.8	68.3	92.5	73.2	71.2
Of those revascularized, probability of receiving CABG (%)	92.8	90.6	84.3	83.8	90.0	83.0
**3 -vessel disease (%,n)**	**21.3 (996)**	**30.8 (614)**	**36.2 (438)**	**9.4 (161)**	**18.1 (188)**	**26.9 (237)**
Probability of revascularization (%)	82.0	73.5	67.1	75.8	68.1	63.3
Of those revascularized, probability of receiving CABG (%)	44.9	52.3	42.5	50.8	39.8	28.7
**1- or 2-vessel disease (%,n)**	**73.5 (3441)**	**58.1 (1158)**	**46.8 (566)**	**88.3 (1521)**	**77.9 (809)**	**65.7 (579)**
Probability of revascularization (%)	53.6	54.6	53.2	31.6	41.4	46.8
Of those revascularized, probability of receiving CABG (%)	13.5	21.4	28.2	19.8	21.2	21.0
***Mean utility scores (EQ-5D)***						
**Left main disease (n)**	**275**	**273**	**116**	**45**	**57**	**35**
Revascularized (mean, SD)	0.77 (0.26)	0.80 (0.22)	0.78 (0.25)	0.76 (0.25)	0.75 (0.29)	0.87 (0.14)
Medical management (mean, SD)	0.81 (0.21)	0.77 (0.26)	0.86 (0.15)	0.87 (0.14)	0.81 (0.21)	0.70 (0.28)
**3 -vessel disease (n)**	**1094**	**822**	**350**	**185**	**209**	**181**
Revascularized (mean, SD)	0.80 (0.22)	0.81 (0.21)	0.82 (0.21)	0.82 (0.17)	0.80 (0.22)	0.80 (0.22)
Medical management (mean, SD)	0.80 (0.21)	0.79 (0.24)	0.81 (0.22)	0.80 (0.22)	0.81 (0.24)	0.84 (0.22)
**1- or 2-vessel disease (n)**	**3056**	**1070**	**357**	**1181**	**667**	**339**
Revascularized (mean, SD)	0.81 (0.23)	0.82 (0.23)	0.83 (0.24)	0.81 (0.21)	0.84 (0.20)	0.85 (0.20)
Medical management (mean, SD)	0.81 (0.23)	0.81 (0.23)	0.80 (0.22)	0.82 (0.22)	0.80 (0.24)	0.81 (0.21)

* All inputs are calculated from the Alberta Provincial Project for Outcomes Assessment in Coronary Heart disease database; a prospective on-going registry of all patients undergoing cardiac catheterization.

**calculated based on patients undergoing catheterization for stable angina dying within 3 days

Ethics approval was obtained from the University of Calgary Conjoint Ethics and Research Board. Three patient cohorts from APPROACH were used to inform our economic evaluation [7]. Patients undergoing catheterization between 2004 and 2005 (N = 11,527) were used to estimate revascularization rates separately for ACS and non-ACS patients. This cohort was selected to accurately reflect recent practice patterns. A larger patient cohort (1995-2005, N = 78,881) was selected to estimate long-term survival, based on ACS-indication, age, sex, coronary anatomy, and treatment received. A subset of this cohort with coronary artery disease is followed forward using a mail-out survey, including a quality of life questionnaire, at 1 year. Only those with complete quality of life data at 1 year were used to calculate HRQOL (N = 10,312). Costing data on post-procedural follow-up care and costs of readmission and subsequent physician visits, meanwhile, were not available for either of the recent patient cohorts, but were available for a 1995-1997 APPROACH cohort (N = 17379) of catheterized patients [11,12].

#### 2. Catheterization rate and detected disease severity

The 2005 population catheterization rate for Alberta was 496 per 100,000 adults over 20, with a range of population catheterization rates of 347 to 542 for Alberta's nine health regions [10]. A theoretical maximum catheterization rate was chosen to reflect, based on expert opinion, the highest possible catheterization rate (2,000 per 100,000). The likelihood of undergoing catheterization is calculated by dividing the number of catheterizations associated with a given population rate by number of catheterizations associated with the maximum population rate.

To calculate the yield of left main, 3-vessel and 1-or 2-vessel disease, a previously published analysis by Graham et al. was replicated using current data (1995-2006) [6]. Briefly, using a hierarchical mixed effects linear model with a random effect for health region, a single weighted line was plotted to reflect the linear relationship between catheterization rate and disease detection rate. This resulted in a linear equation where the slope represents the additional yield of disease case per unit increase in population catheterization rate. A quadratic term was included in the model to test for evidence of a plateau in the yield of high-risk CAD. The quadratic term was non-significant indicating no evidence of a plateau in the high-risk CAD cases detected at the highest observed catheterization rate. The high-risk model demonstrated that males have a higher yield of high risk disease than do females. In males, approximately 1 in 3 catheterizations yields a patient with left main or 3-vessel disease care whereas in females the ratio is approximately 1 in 5.

#### 3. Event rates and long-term survival of those catheterized (Table 1, Figure 2)

**Figure 2:**
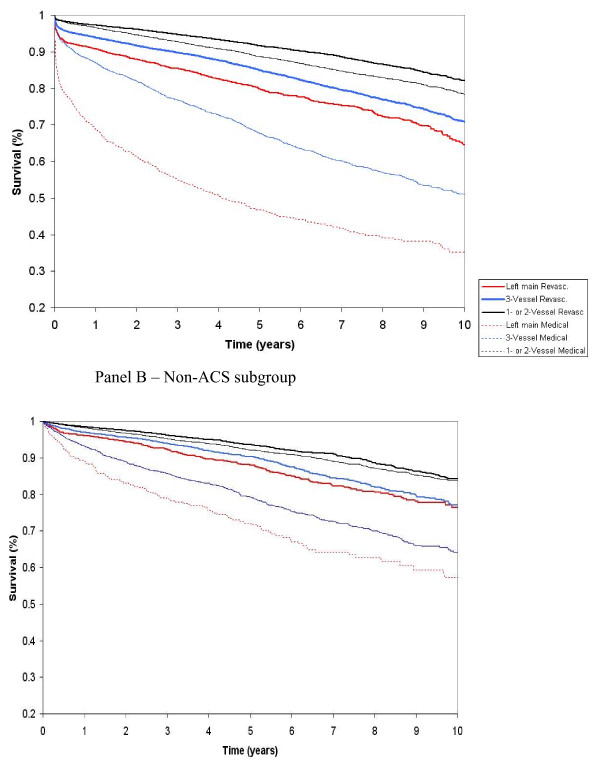
**Observed 10-year survival of patients undergoing cardiac catheterization in Alberta, categorized by ACS, disease and treatment subgroup (data is from the Alberta Provincial Project for Outcomes Assessment in Coronary Heart disease database; a prospective on-going registry of all patients undergoing cardiac catheterization)**.

Rates of PCI and CABG were calculated by ACS, disease (i.e. left main disease, 3-vessel disease, 1-to-2 vessel disease, or normal/near normal coronaries), age and sex subgroup of patients undergoing cardiac catheterization. Although the majority of patients with left main and 3-vessel disease undergo revascularization, some patients are managed medically (presumably due to patient preference, severity of comorbid illness, or coronary anatomy precluding adequate revascularization). Patients with left main disease are managed predominantly with CABG, whereas patients with 3-vessel disease appear to undergo PCI or CABG equally. For the "standard care comparator strategy", we assumed that the same proportion of patients would be eligible to undergo revascularization (i.e. PCI, CABG) as those who currently receive catheterization.

Long-term survival data for the larger cardiac catheterization cohort were available for 10 years. Kaplan-Meier plots were produced by disease and ACS subgroup for those revascularized and then separately for those medically managed. Those revascularized with left main or 3-vessel disease had better long-term survival than those managed medically although this may result from many factors including patient selection, rather than just the treatment received. However, patients with 1- or 2-vessel disease have similar survival regardless of whether they are revascularized or medically managed, particularly in the non-ACS subgroup.To account for increasing age-related mortality, after 10 years, the hazard ratios for each of the patient subgroups were multiplied by the age-specific increment in mortality risk of the Canadian population [13].

#### 4. Health-Related Quality of life (Table 1)

HRQOL estimates were determined for the cohort of APPROACH patients noted above using self-reported Euroqol (EQ-5D) index scores completed one year after catheterization [14]. Mean utility scores were calculated for patients by age, sex, extent of coronary disease and revascularization status. We assumed that quality of life scores for those revascularized and those medically managed persisted for the patient's lifetime though this was varied in sensitivity analysis.

For patients with high-risk coronary disease who are not catheterized (and thus do not receive revascularization), evidence from the MASS-2 trial which compared CABG, PCI and medical management in patients with multi-vessel coronary disease was used [15]. SF-36 scores, a measure of general HRQL, was measured in the MASS-2 study; patients who were revascularized reported higher SF-36 scores at 6-months and 1-year compared to those who received medical management alone. The SF-36 scores were converted to a utility score using a published, validated algorithm [16-18]. The difference between the scores of those revascularized and those medically managed (0.03) was subsequently subtracted from patients potentially eligible for revascularization with undetected left main and 3-vessel disease who by definition received only medical management. There is no evidence to suggest a HRQOL benefit with revascularization in 1- or 2-vessel disease. Thus, the mean utility score in this group of patients for those potentially revascularized (not undergoing catheterization) was assumed to be the same as those undergoing revascularization.

#### 5. Costs (Table 2)

**Table 2:** Total annual healthcare costs* (2006 Canadian $), by treatment and age subgroup (N = 17379)**

	Medically managed			PCI			CABG		
	Under 65	65-75	Over 75	Under 65	65-75	Over 75	Under 65	65-75	Over 75
**Left main disease (n)**	**n = 98**	**n = 117**	**n = 70**	**n = 43**	**n = 35**	**n = 11**	**n = 330**	**n = 382**	**n = 150**
Year 1 (mean, 95% CI)	2166 (710-3622)	4312 (2941-5683)	3756 (2112-5401)	5913 (3749 - 8077)	4597 (2428-6765)	4123 (946-7300)	10990 (9581-12399)	15342 (13486-17198)	11884 (9387-14381)
Year 2 (mean, 95% CI)	1793 (488-3100)	3806 (2265-5347)	5777 (2835-8719)	3580 (1014-6146)	5536 (893-10179)	7739 (0-16190)	1414 (1045-1782)	2041 (1656-2426)	2385 (1626-3143)
Year 3 (mean, 95% CI)	1641 (380-2903)	3527 (1765-5290)	2703 (938-4468)	1332 (256-2407)	1680 (543-2817)	3180 (0-8731)	1392 (1000-1784)	2413 (1708-3117)	1909 (1375-2444)
**3 -vessel disease (n)**	**n = 684**	**n = 631**	**n = 352**	**n = 646**	**n = 387**	**n = 186**	**n = 881**	**n = 697**	**n = 256**
Year 1 (mean, 95% CI)	2467 (2038-2896)	3880 (3289-4470)	4121 (3329-4913)	5139 (4530-5748)	6617 (5612-7621)	5545 (4504-6585)	13539 (12463-14615)	17444 (16006-18882)	18291 (15613-20969)
Year 2 (mean, 95% CI)	3348 (2568-4127)	4552 (3782-5322)	3394 (2580-4208)	2503 (1991-3014)	3217 (2565-3869)	3706 (2457-4955)	1586 (1334-1839)	2503 (2124-2882)	2943 (2163-3722)
Year 3 (mean, 95% CI)	2790 (2188-3392)	3495 (2811-4179)	3235 (2375-4095)	2150 (1700-2601)	3494 (2759-4229)	2568 (1901-3234)	1412 (1166-1658)	2319 (1959-2680)	2458 (1876-3040)
**1- or 2-vessel disease (n)**	**n = 4132**	**n = 1773**	**n = 670**	**n = 2495**	**n = 1063**	**n = 388**	**n = 490**	**n = 301**	**n = 111**
Year 1 (mean, 95% CI)	2616 (2375-2857)	3866 (3398-4334)	4420 (3758-5081)	4351 (4046-4657)	5095 (4617-5574)	5792 (4955-6630)	13937 (12369-15505)	17732 (15482-19982)	16910 (13050-20771)
Year 2 (mean, 95% CI)	1808 (1635-1980)	2988 (2644-3333)	3624 (2965-4283)	1739 (1553-1925)	2691 (2350-3031)	2797 (2241-3353)	1865 (1468-2260)	2400 (1832-2969)	3138 (1670-4605)
Year 3 (mean, 95% CI)	1658 (1494-1821)	2883 (2570-3197)	3144 (2513-3774)	1487 (1328-1647)	2708 (2321-3096)	2903 (2236-3570)	1660 (1198-2123)	2110 (1656-2564)	2928 (1375-4481)

*Includes all subsequent hospitalization, physician, ambulatory care, home care and drug costs.

* *All inputs are calculated from the Alberta Provincial Project for Outcomes Assessment in Coronary Heart disease database; a prospective on-going registry of all patients undergoing cardiac catheterization.

For those undergoing catheterization, data on costs were obtained from Alberta Health and Wellness (the sole payer for hospitalization and physician care in Alberta) for the 1995-1997 APPROACH cohort. The costs associated with catheterization, CABG and PCI were noted to be $2048, $17,958 and $7,927 respectively, including procedure costs, and associated hospitalization and physician costs. Subsequent annual costs were obtained for hospitalization, ambulatory care, home care, physician claims and medication costs until March 2001. Ambulatory care costs were restricted to cardiac care. Costs were estimated on an annual basis from the date of first catheterization, and were adjusted to 2006 dollars using a yearly health sector inflation factor [19]. Given that costs were observed to be relatively constant between years 2 and 3, regardless of what cardiac procedure treatments patients had received, we assumed that yearly costs remained constant after three years.

There is uncertainty whether revascularization impacts the long-term costs of patients with coronary disease compared with medical management. However, the RITA-2 trial, a trial comparing PCI to medical management in patients with angina, reported similar annual costs of care over a 3-year period between the treatment strategies [20]. Thus, we assumed no difference in annual costs of care between those undergoing catheterization and those not.

#### 6. Efficacy of revascularization (Table 3)

**Table 3:** Relative risk of death associated with medical management compared to revascularisation

Non-ACS (by disease)				
Time Period	Left main	3-vessel	2- and 1-vessel	Source
0-5 years (95% CI)	2.33 (1.27-4.60)	1.59 (1.21 - 2.14)	1.27 (0.88-1.85)	[5]
5-10 years (95% CI)	0.74 (0.46-1.21)	1.00 (0.87 - 1.14)	0.80 (0.70-0.91)	[5]
10 + years (95% CI)	1.00	1.00	1.00	Assumed

**ACS (unavailable by disease)**				
0-2 years	1.47			[22]
2-5 years	0.88			[4]
5+ years	1.00			Assumed

*RR > 1.00 indicates revascularisation more beneficial

In this study, we estimated survival rates based on APPROACH patients with high-risk coronary anatomy who have undergone revascularization, but do not have survival rates for patients with undetected high-risk coronary disease who receive medical management. To estimate the survival of high-risk patients who receive medical management within our model, we used a meta-analysis by Yusuf et al that reported the odds ratio of mortality associated with CABG compared to medical therapy for stable (non-ACS) patients with high-risk coronary disease, by disease severity, for two time periods; 0-5 years and 0-10 years [5]. Using a validated formula, we converted both odds ratios into a relative risks and imputed the 5-10 year risk [21]. To calculate the survival of those not catheterized, we applied the inverse relative risk for both the 0-5 year and 5-10 year relative risk to the observed long-term survival data of APPROACH high-risk patients who were revascularized by disease severity. The relative risk of death, based on Yusuf et al., of those medically managed as opposed with revascularization is 2.33 for patients with left main disease, 1.59 for 3-vessel disease and 1.27 for 1- or 2-vessel disease for 0-5 years post-revascularization [5]. During the 5-10 year window after revascularization, the risk of death for those medically managed appears lower than those who underwent revascularization (RR of death with medical management compared to revascularization of 0.74 for left main, 1.00 for 3-vessel and 0.80 for 1- or 2-vessel disease)

Recognizing the trials included in Yusuf's meta-analysis excluded ACS patients, a second baseline analysis applied the relative risk from the FRISC-II study to the ACS subgroup [4,22]. The FRISC-II trial randomized patients to an early catheterization strategy compared to a conservative, symptom-guided catheterization strategy. Thus, FRISC-II did not report the efficacy of revascularization, per se, as patients in both arms could undergo intervention. However, it appeared as though the benefit seen from early catheterization is due to the increased use of early revascularization. Two separate relative risks were applied; 0-2 years and 3-5 years based on the 2 publications of FRISC-II [4,22]. Unlike Yusuf's meta-analysis for CABG in non-ACS patients, the FRISC-II results are not reported by disease subgroup. Thus, the same relative risk had to be applied across all coronary disease subgroups. While not optimal, given that there are no RCTs reporting on the efficacy of revascularization compared with medical management in ACS patients, this analysis serves as our best estimate of the efficacy of increasing catheterization rates in ACS patients. The resulting RR from the FRISC-II trial (ACS patients) is 1.47 for 0-2 years and 0.88 for 3-5 years post-revascularization [4,22].

### Economic Analysis

A healthcare payer perspective was adopted. Costs and outcomes were discounted using an annual discount rate of 5 percent [5,23]. SAS, version 9.1, was used for all data analysis; all economic modelling was done in Treeage Pro 2009.

### Scenario and Sensitivity Analyses

All sensitivity analyses were completed for the ACS and non-ACS subgroup separately. The impact of various high-risk case yields was explored. We considered an ACS and non-ACS specific yield rate. Subsequently, for males, the yield was varied from 1 case detected in 2 to a minimum of 1 case per 4 catheterizations (base case: 1 case detected in 3 catheterizations). For females, a maximum yield of 1 case detected in 2 and a minimum 1 case detected in 7 catheterizations was considered (base case: 1 case detected in 5 catheterizations).

The base case utility for patients treated medically or with PCI or CABG was estimated from the EQ-5D utility scores taken from the APPROACH cohort. Given its potential lack of sensitivity to changes in HRQOL, in sensitivity analysis, a disease-specific measure (the Seattle Angina Questionnaire) was also considered. The Seattle Angina Questionnaire is a measure of quality of life (not utility), and thus is not ideal for use in the context of an economic evaluation. However, given there are no cardiac disease-specific utility instruments, we applied the quality of life scores from the Seattle Angina Questionnaire (which range from 0 to 1) to understand how different utility estimates might impact the cost per QALY. The resulting utility estimates for those revascularized and those medically managed respectively were 0.87 and 0.71 for left main, 0.83 and 0.72 for 3-vessel disease and 0.80 and 0.78 for 1- and 2-vessel disease. We also varied the relative risk associated with revascularization by +/- 25%, and considered a scenario where a constant survival benefit associated with revascularization was assumed over a 10 (rather than five) year time period. Lastly, procedural costs, and yearly costs of care were increased and decreased to explore the impact of changes within these variables on the resultant cost per QALY.

### Probabilistic Sensitivity Analysis

To estimate the overall uncertainty in the model, we completed a Monte Carlo Simulation for the ACS and Non-ACS models using the Yusuf estimates of efficacy. Normal distributions were used for relative risks, utility estimates and the high-risk yield. Gamma distributions were used for all cost estimates. Incremental cost-effectiveness scatterplots, with 95% confidence ellipses, were produced to visually assess the uncertainty in the model. In addition, cost-effectiveness acceptability curves were plotted to demonstrate the likelihood that an alternative is cost-effective at a specified willing-to-pay threshold.

## Results

### Cost-effectiveness of Increasing Catheterization Rates

Table 4 presents the cost-effectiveness of increasing the population rate of cardiac catheterization. On average, the incremental cost per additional catheterization done is $5,270. The incremental effectiveness per additional catheterization performed is 0.203 QALYs gained, or 74 healthy days. As such, for every additional potentially eligible patient undergoing cardiac catheterization, increasing the catheterization rate is associated with a cost per QALY gained of $26,470.

**Table 4:** Cost per QALY gained with increased population catheterization rate compared to the current catheterization rate, overall and by subgroup

Strategy	Incremental Costs per catheterization($)	Incremental Effectiveness per catheterization (QALY)	Incremental cost-effectiveness ratio ($ per QALY)
**Overall**	**5,270**	**0.203**	**26,470**
*ACS subgroup - Yusuf RR*	*5,329*	*0.226*	*23,559*
Males	6,047	0.245	24,725
Females	3,801	0.187	20,320
Age < 65	4,733	0.192	24,680
Age 65-75	6,939	0.244	28,495
Age > 75	4,976	0.301	16,538
*ACS subgroup - FRISC-II RR*	*5,136*	*0.163*	*31,438*
Males	5,838	0.176	33,158
Females	3,645	0.136	26,720
Age < 65	4,622	0.147	31,466
Age 65-75	6,645	0.169	39,405
Age > 75	4,664	0.200	23,360
*Non-ACS subgroup - Yusuf RR*	*5,219*	*0.163*	*32,107*
Males	5,686	0.193	29,465
Females	4,228	0.100	43,167
Age < 65	4,534	0.143	31,731
Age 65-75	6,307	0.187	33,748
Age > 75	5,976	0.204	29,286

When an ACS scenario is considered, applying the Yusuf relative risks [5] (that were derived on stable angina patients), the cost per QALY is $23,559 (Table 4). Within the ACS subgroup, females and those over 75 years of age have the lowest cost per QALY at $20,320 and $16,538 respectively. When the FRISC-II relative risk [4,22] is applied to the ACS subgroup, the cost per QALY increases to $31,438, reflecting the smaller benefit modelled compared to the Yusuf work (Table 4).

### Sensitivity and Scenario Analyses

Table 5 summarizes the various sensitivity analyses completed. As expected, when the yield of high-risk cases is lower, the cost per QALY is less attractive. The cost per QALY is also sensitive to variations is the relative risk of survival associated with revascularization. Of note, when the Yusuf relative risk from 0-10 years is decreased by 25% (the RR after year 10 remains 1.0) as might be plausible if the additional high risk patients that are identified benefit less from revascularization, increasing the catheterization rate becomes more costly and less effective (dominated) than maintaining the current catheterization rate. Using the Seattle Angina Questionnaire to measure HRQOL, an instrument that results in more pronounced HRQOL differences between receiving revascularization compared to medical management, the cost per QALY is more attractive in all subgroups.

**Table 5:** Sensitivity analyses

Parameter	Incremental cost-effectiveness ratio ($ per QALY)		
	**ACS** **(Yusuf RR)**	**ACS** **(FRISC-II RR)**	**Non-ACS**

*Base case*	*$23,559*	*$31,438*	*$32,107*
ACS specific yield (2.6:1 for males, 3.4:1 for females)	$22,187	$30,768	
Non-ACS specific yield (2.5:1 for males, 5:1 for females)			$29,593
Disease yield for males (cost per QALY for male subgroup)			
Decreased to 4:1 catheterizations per high risk case detected	$28,335	$34,139	$34,673
Increased to 2:1 catheterizations per high risk case detected	$21,973	$32,167	$25,466
Disease yield for females (cost per QALY for female subgroup)			
Decreased to 7:1 catheterizations per high risk case detected	$22,485	$27,626	$51,189
Increased to 3:1 catheterizations per high risk case detected	$17,410	$24,944	$32,390
Relative risk of death associated with medical management compared to revascularisation			
Increase by 25% (revascularization more beneficial)	$13,300	$18,717	$17,884
Decrease by 25% (revascularization less beneficial)	Dominated	$196,414	Dominated
Use of Seattle Angina Questionnaire disease specific quality of life measure	$20,975	$27,575	$26,264
Procedural Costs			
Increase by 50%	$30,631	$41,228	$40,540
Decrease by 25%	$16,486	$21,648	$23,674
Costs of care			
Increase by 50%	$21,193	$27,577	$31,294
Decrease by 25%	$24,741	$33,368	$32,513
Discount rate			
No discounting	$12,324	$15,388	$12,672
3% discount rate	$19,300	$25,307	$24,276
6% discount rate	$25,617	$34,389	$35,971

### Probabilistic Sensitivity Analysis

**Figure 3:**
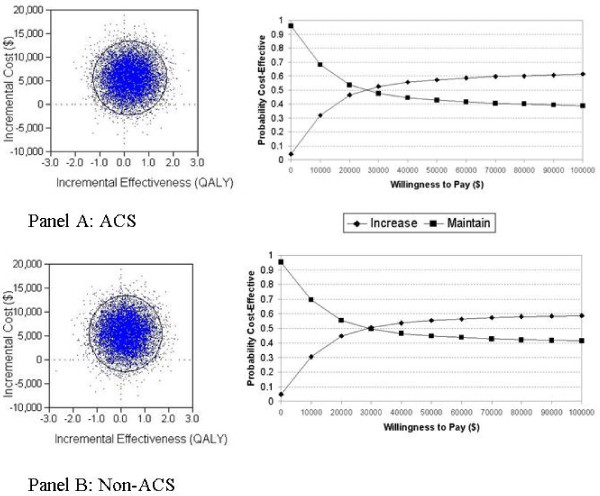
**Incremental cost-effectiveness scatterplots and cost-effectiveness acceptability curves, by ACS/Non-ACS subgroup**.

Figure 3 presents the incremental cost-effectiveness scatterplots. There are points in all four quadrants indicating significant uncertainty in the results. This is largely due to the wide confidence intervals for the relative risks of death which cross 1.0 indicating both benefit and harm associated with revascularization. For the ACS subgroup, a strategy of increasing the catheterization rate is associated with a cost per QALY of less than $50,000, a willingness-to-pay threshold commonly proposed as reasonable value for money [24], in 56.3% of the simulations. However, 34.1% of the simulations result in a situation where increasing the catheterization rates is less effectiveness and more expensive than maintaining the current catheterization rate; a situation where a policy should not be adopted. The cost-effectiveness acceptability curve show that at a willingness-to-pay threshold of $50,000, the probability that a strategy of increased catheterization is cost-effective is 57%. If the willingness-to-pay threshold is increased to $100,000, the probability increases slightly to 61%. If the willingness-to-pay threshold is less than approximately $25,000 maintaining the current catheterization rate is the most attractive strategy. The results are similar for the Non-ACS subgroup.

## Discussion

In many health care systems, the availability of coronary catheterization facilities and access to such procedures is viewed as a key indicator of the "health" of a health care system [25]. As a result, the dialogue surrounding issues of access to invasive cardiac procedures is politically charged and loudly debated among providers, health system decision makers, and the general public. Yet, such debate over the availability of procedures is not fully informed, because arguments for more or less procedures often overlook the economics of providing increased access through the provision of more procedures. It is in this context that we conducted this economic evaluation, with the objective of providing valuable economic data that can inform the planning of cardiac catheterization and revascularization resources at a population level.

We found that a strategy of increasing the population catheterization rate overall in eligible patients is associated with a cost per QALY of $26,470, with cost per QALY estimates ranging from approximately $23,000 to $32,000 depending on the subgroups of greatest interest. Our results appear sensitive to the effectiveness of revascularization among the additional high risk patients that are identified, and further information on this variable would be ideal. In addition, probabilistic sensitivity analysis demonstrates significant model uncertainty with scatter falling within all four quadrants of the ICER scatterplots. In Canada, $20,000-$100,000 is often cited as a range which could be considered good value for money [26]; a range within which the cost-effectiveness acceptability curve shows probabilities of 40-61% of increasing the catheterization rates being cost-effective. Other countries have cited different threshold values (₤30,000 in the UK and $50,000 in the US [24,27]).

Our economic evaluation contributes new information. An economic analysis of the RITA-2 trial, which included patients eligible for both PCI and medical management, found that the upfront costs of PCI are not recouped within 3 years in cost-savings compared to medical therapy [20]. Economic analysis of the RITA-3 trial, which assessed a strategy of early catheterisation versus conservative management in ACS patients found a cost per QALY of ₤12,000 in this high-risk group [28]. A cost-effectiveness study of the FRISC-2 trial in ACS patients, with a short-term perspective, reported a cost per death avoided of 1,404,000 Swedish Krona (CAN $ 222,439) associated with an early catheterization strategy compared to medical management [29]. Interpreting a cost per death avoided is difficult in the context of other interventions that are expressed as a cost per QALY over a lifetime horizon. Results from the COURAGE trial, a contemporary trial comparing PCI to medical therapy revealed a cost per QALY of US $206,229, likely due to this study's inclusion of relatively low risk, stable patients [30]. Lastly, Griffin et al reported a cost per QALY of ₤19,000 for CABG compared to medical management after 6 years of follow-up in patients deemed eligible for both PCI and CABG [31]. Our analysis, however, differs from these prior studies and also expands on them. First, we incorporate quality of life benefits associated with revascularization recognizing that the benefit associated with revascularization may be broader than mortality, thus allowing for a cost per QALY to be calculated. Our analysis also considers a lifetime horizon over which additional benefit is accrued. In addition, our study includes older patients with more severe disease, where more benefit may be seen.

Although increasing catheterization rates appears potentially attractive when considering the cost per QALY estimate in isolation, it is important to judge it against the cost-effectiveness of other candidate therapeutic innovations (e.g., new cancer chemotherapies, or a population-based vaccination strategy) that may be under consideration by health system decision-makers at any given moment in time. Our cost-effectiveness estimates, while useful, are thus only part of the answer to such challenging societal and health funding decisions.

There are some caveats and limitations to our analysis. The primary benefit of catheterization and subsequent revascularization in high-risk patients is increased survival. Our analysis used a relative risk of survival for revascularization in high risk patients from a meta-analysis of trials done nearly 20 years ago [5]. While this information could be criticized as out of date, these studies have been the only RCTs that have compared medical management to revascularization in high-risk disease patients (left main and 3-vessel) and these studies continue to guide care of these patients. It could be argued that medical management has improved greatly since these trials were completed and if the survival of medically-managed patients is better now, the advantage of revascularization could be lower, with a resultant less attractive cost per QALY. However, it could also be argued that patient who are managed with revascularization also receive medical management (i.e. better blood pressure control and better management of hyperlipidemia), so the relative advantage attributable to revascularization may still be applicable. We also applied more recent evidence from the FRISC-II trial and found generally similar results to those produced by the Yusuf relative risks [4,5,22]. As noted, our results are sensitive to the estimate used from the effectiveness of revascularization among the additional high risk patients that are identified; accordingly, further information on this variable should be sought. Our use of these efficacy estimates in our economic modelling underlines the fact that our collective knowledge of the benefit of revascularization is somewhat outdated for stable angina patients (as per the Yusuf meta-analysis) and indirect for ACS patients (from FRISC-II).

The recent COURAGE trial also warrants mention in the context of considering efficacy estimates for revascularization in our economic evaluation [32]. That trial compared contemporary optimized medical therapy vs. PCI in patients with stable angina and varying degrees of coronary disease. The study revealed no difference between groups for the composite endpoint of mortality or MI, but a slightly lower risk of mortality in patients undergoing PCI (relative risk of 0.87). The lack of coronary anatomy-specific relative risk estimates, the upfront exclusion of patients with high risk clinical profiles, and the study's focus on only stable angina patients are all factors that prevent us from using the COURAGE trial as an efficacy input to our economic model.

The feasibility of increasing the catheterization rate is another component of the decision that is not directly addressed by our analysis. Our analysis assumes that additional catheterizations would be performed within established infrastructure and existing manpower (and as such that additional capital resources are not required). If the current facilities are operating at full capacity, then additional facilities would be required to increase the number of catheterizations. This would require additional capital investment which would increase the cost per additional catheterization done potentially making the cost per QALY less economically attractive. Our analysis addresses whether increasing catheterization rates provides reasonable value for money but does not address the optimal strategy for increasing catheterization rates. Given that low catheterization rates may have many reasons and considering the differences among health care systems, local health regions will have to consider the most appropriate strategy for their local health care environment. Finally, our work does not consider the use of non-invasive testing. Its potential proliferation in coming years may impact the cost-effectiveness of catheterization as patient selection and risk-stratification change. Future work could compare the cost-utility of catheterization to non-invasive imaging in a well-defined patient population. These caveats and limitations notwithstanding, our study adds new information that can inform the population-based planning of cardiac catheterization and revascularization services.

## Conclusion

Increasing the population catheterization rate is associated with a cost per QALY of $26,470. However, although the basecase estimate generally supports a strategy of increasing cardiac catheterization rates within eligible patients, conclusions about the cost-effectiveness of increasing the catheterization rate are inconclusive due to significant model uncertainty associated with the efficacy of revascularization. In addition, funding decisions also require the careful strategic consideration of other efficacious health care interventions and the accompanying opportunity costs.

## Competing interests

The authors declare that they have no competing interests.

## Authors' contributions

FC, BM, and WG contributed to the conception and design, analysis and interpretation of data. All authors contributed to the drafting and revision of the article for critically important intellectual content. All authors approved the final version of the manuscript.

## Pre-publication history

The pre-publication history for this paper can be accessed here:

http://www.biomedcentral.com/1472-6963/11/324/prepub
